# Achromatic Petal‐Shaped Beam Generation and Information Encoding Based on Perfect Vortex Beam

**DOI:** 10.1002/nap2.70133

**Published:** 2026-05-20

**Authors:** Yilong Ge, Yifeng Wei, Jinqin Cui, Ziben Liu, Yang Liu, Bingxue Zhang, Dandan Wen, Peng Li, Sheng Liu, Jianlin Zhao, Dong Li

**Affiliations:** ^1^ Key Laboratory of Light Field Manipulation and Information Acquisition, Ministry of Industry and Information Technology, and Shaanxi Key Laboratory of Optical Information Technology School of Physical Science and Technology Northwestern Polytechnical University Xi'an China

**Keywords:** achromatic, metasurface, Pancharatnam‐Berry phas, perfect vortex beam, petal‐shaped beam

## Abstract

Based on the successful generation of petal‐shaped beams with controllable topological charges (TCs), sizes, and rotation angles, this work employs a spatially multiplexed all‐dielectric TiO_2_ transmissive array to achieve focused petal‐shaped beams at 480 nm, 530 nm, and 630 nm wavelengths, converging to the same focal plane through numerical simulation. We propose a new encoding scheme with different rotation angles and spot numbers, based on the characteristic doughnut‐shaped intensity profile with certain TCs and an incremental rotational modulation, thereby enhancing the capacity of information transmission. Leveraging the high spatial multiplexing capability of all‐dielectric metasurfaces and the flexible tunability of petal‐shaped beams, we present the design and implementation of an achromatic petal‐shaped beam platform operating across multiple wavelength bands. These results lay the groundwork for expanding the applications of petal‐shaped beams in information processing and optical micromanipulation.

## Graphical abstract

Based on petal‐shaped beams with controllable topological charge, size, and rotation angle, this work employs a spatially multiplexed all‐dielectric TiO_2_ transmissive array to numerically achieve achromatic petal‐shaped beams at three wavelengths: 480 nm, 530 nm, and 630 nm. Leveraging their unique intensity distribution and rotational characteristics, a novel encoding scheme is proposed to effectively enhance information transmission capacity.

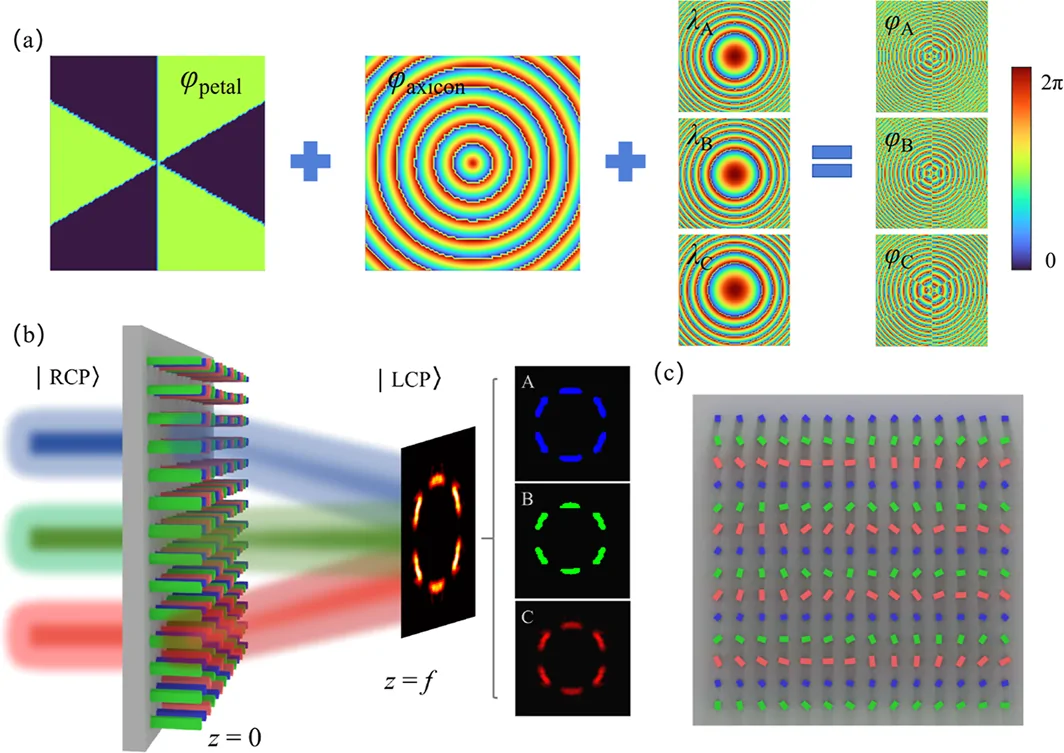

## 1 Introduction

Optical vortex beams have attracted significant attention in scientific and communication fields due to their orbital angular momentum (OAM) properties [1, 2]. However, the dependence of conventional vortex beam ring radius on topological charge results in mode size discrepancies that prevent effective spatial superposition of beams with different TCs, consequently limiting their application potential in areas such as multiplexed communications [3, 4] and optical manipulation [5, 6]. Within this context, Ostrovsky et al. proposed the concept of the perfect vortex beam (PVB) [7], which exhibits a topological‐charge‐independent annular radius distribution and has attracted extensive research attention. Current established PVB generation techniques include axicon lenses [8], spatial light modulators [9], interferometers [10], and digital micromirror devices [11]. However, these conventional systems suffer from a critical limitation: stringent optical assembly tolerance [12], where even minor displacements can introduce wavefront distortions and degrade beam quality.

As emerging two‐dimensional planar optical devices, metasurfaces have garnered substantial interest in optics due to compact structure, robust wavefront manipulation capabilities, and high design flexibility [13, 14, 15, 16]. The development of three‐stage metasurface systems incorporating axicon, spiral, focusing phases [17], and integrated single‐metasurface designs [18] has opened possibilities for efficient PVB generation. PVBs with multiple TCs have been extensively generated using plasmonic [19, 20, 21] and dielectric metasurfaces [22, 23, 24, 25]. As PVBs find expanding applications in optical communications, information storage, and particle manipulation [26, 27, 28, 29, 30, 31, 32, 33], topological charge identification limitations have become increasingly prominent. Conventional detection methods such as interferometry and deep learning‐assisted diffraction pattern recognition rely on complex external optical systems or post‐processing computation [34, 35]. This shifts identification from the optical to electronic/digital domain, introducing additional burdens in system size, cost, complexity, and processing latency. Furthermore, the dispersive nature of dielectric materials causes metasurfaces to produce distinct phase responses at different wavelengths, leading to significant phase distortion and functional degradation when operating at nondesign wavelengths. To address chromatic aberration, researchers have developed various compensation approaches. Capasso et al. demonstrated an achromatic silicon metasurface achieving diffraction‐limited focusing at 1300/1500/1800 nm [36]. Subsequently, to further extend the operational bandwidth into the visible spectrum and enhance device performance, the team innovatively employed titanium dioxide (TiO_2_) as the metasurface material [37]. In the same year, Faraon et al. realized co‐focusing of 950 and 1550 nm beams through spatially multiplexed polarization‐insensitive metasurfaces [38]. The above research demonstrates that by dividing the aperture of a single metasurface into row‐based functional regions, each designed to focus a specific wavelength while maintaining the same focal length, the device can achieve achromatic focusing simultaneously at multiple discrete wavelengths. Furthermore, combined with the high‐aspect‐ratio TiO_2_ nanopillars, which possess both high refractive index and low loss, the device provides the necessary phase control capability across the visible band. To achieve the transition from multi‐wavelength to broadband operation, Capasso et al. introduced wavelength‐dependent optimization parameters employing particle swarm algorithms to realize efficient reflective achromatic metasurfaces [39]. Subsequently, Wang et al. leveraged GaN properties to achieve transmissive achromatic metalenses covering 400−600 nm [40]. Chen et al. accomplished simultaneous dispersion compensation and phase control across 460−700 nm through co‐optimization of propagation and geometric phases [41]. Yao et al. developed a multi‐nanorod nonlocal plasmonic integrated resonance unit design based on phase‐compensated resonance control, achieving achromatic focusing across 400–660 nm spectral range [42]. Although optimization algorithms can explore complex design spaces in achromatic metasurface design, they face limitations such as slow convergence, high computational cost, and initial parameter sensitivity. In contrast, spatial multiplexing allocates wavelength‐specific wavefront control to decoupled meta‐atoms, providing stable performance and low crosstalk. Its straightforward workflow enables multi‐wavelength control without iterative optimization.

In this work, we propose and demonstrate a scheme for generating petal‐shaped beams using a single all‐dielectric achromatic metasurface, the metasurface is composed of TiO_2_ nanopillars acting as deep‐subwavelength half‐waveplates, and able to provide the desired geometric phase profile to an incident circularly polarized light for the generation of petal‐shaped beams. We first systematically investigated the generation mechanisms of petal‐shaped beams with different topological charges, sizes, and rotation angles, where the beams preserve the fundamental “perfect” characteristic of conventional PVBs through radius invariance with topological charge while simultaneously forming topological‐charge‐dependent petal‐shaped intensity distributions in the focal plane. By carefully optimizing the geometric parameters and spatial arrangement of the metasurface nanostructures, we mitigated the chromatic dispersion inherent under broadband operation. This approach achieved stable generation of petal‐shaped beams across the visible spectrum (480 nm, 530 nm, and 630 nm), verifying the effectiveness of the achromatic design. Based on these results, we demonstrated the feasibility of an angle‐incremental encoding scheme that utilizes petal‐shaped beams unique properties, including tunable rotation angle and controllable number of optical spots.

## 2 Design and Theory

The generation of PVBs using conventional optical components typically employs spiral phase plates, axicon lenses, and Fourier‐transform lenses, with the transverse electric field distribution expressed as follows:

(1) $$E\left(r,\theta \right)=i^{l-1}\frac{\omega_{g}}{\omega_{0}}\exp\left(il\theta \right)\exp\left(\frac{-{\left(r-R\right)}^{2}}{\omega_{0}^{2}}\right)$$

Where, *l* denotes the topological charge number, *ω*_*g*_ represents the waist radius of the input Gaussian beam, *ω*_0_ corresponds to the waist radius of the Gaussian beam at the back focal plane, *R = krf/k* defines the radius of the PVB, *k*_*r*_ is the radial wave vector, *k* is the wave vector in free space, and *f* is the focal length of the Fourier‐transform lens.

For metasurface implementation, the required phase profile can be decomposed into three components: the spiral phase, the axicon phase, and the lens phase, expressed as follows:

(2) $$\begin{array}{l} \varphi_{\text{spiral}}\left(r,\theta \right)=l\cdot \theta \\ \varphi_{\text{axicon}}\left(r,\theta \right)=-\frac{2\pi }{\lambda }r\cdot NA\\ \varphi_{\text{lens}}\left(r,\theta \right)=-\frac{2\pi }{\lambda }\left(\sqrt{r^{2}+f^{2}}-f\right) \end{array}$$

where, *NA* denotes the numerical aperture of the axicon lens, *f* is the focal length of the Fourier‐transform lens. Alternatively, replacing the conventional spiral phase with a Laguerre–Gaussian beam also enables efficient generation of PVBs. When the Laguerre–Gaussian beam interacts with an axicon lens, it transforms into a Bessel–Gaussian beam through precise spatial phase superposition, achieving structural conversion of the beam profile. Subsequently, the Bessel–Gaussian beam undergoes Fourier transformation through a lens, ultimately forming a petal‐shaped beam with predefined topological charge characteristics. This approach preserves the fundamental property of PVBs, specifically the independence of ring radius from topological charge, whereas the introduction of Laguerre–Gaussian beam significantly enhances flexibility and tunability in beam generation. It thereby opens new avenues for multifunctional optical field control based on metasurface platforms. To clearly demonstrate its fundamental characteristics, this work considers only the superposition of two Laguerre–Gaussian modes, with the corresponding petal‐shaped phase distribution expressed as follows:

(3) $$\varphi_{\text{petal}\;}\left(r,\theta ,z\right)=\arg\left[e^{i\delta }\left(LG_{p_{1}l_{1}}+LG_{p_{2}l_{2}}\right)\right]$$

For the two modes considered, the radial indices are set as *p*_1_ = *p*_2_ = 0 with an initial phase difference of *δ* = 0. Through superposition with specific parameter combinations, the unique petal‐shaped intensity profile originates from distinctive phase distribution characteristics. When two Laguerre–Gaussian modes carrying different TCs undergo coherent superposition, they generate multiple phase discontinuity lines within the spiral wavefront. These discontinuity lines correspond to dark regions in the intensity distribution. This phase distribution exhibits a special double‐helix structure analogous to the “cake‐cutting” pattern of a normal distribution. By precisely controlling the number and arrangement of these phase discontinuities, a rose‐petal‐like intensity pattern, whose number of petals is governed by the topological charge difference, is reconstructed in the focal plane. Figure 1a illustrates the phase combination process of petal‐shaped beams under different design wavelengths, *λ*_A_ represents 480 nm, *λ*_B_ represents 530 nm, and *λ*_C_ represents 630 nm, its conceptual representation can be expressed as follows:

(4) $$\varphi_{\text{meta}}=\varphi_{\text{petal}}+\varphi_{\text{axicon}}+\varphi_{\text{lens}}$$

**FIGURE 1:**
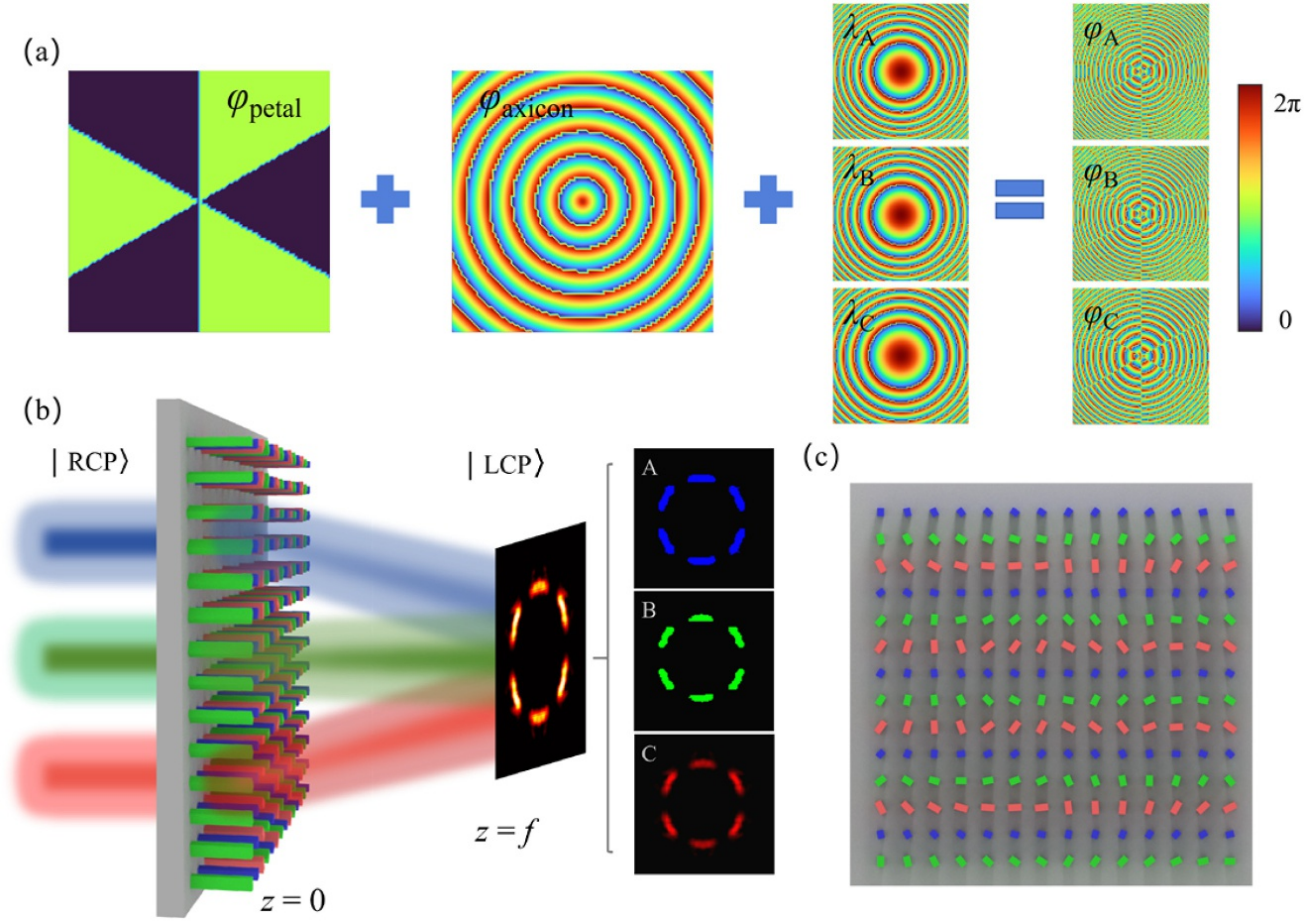
Design of the spatially multiplexed achromatic metasurface. (a) Metasurface phase profile formed by petal‐shaped phases, axicon phase and focusing phase of different design wavelengths. (b) Generation of multi‐wavelength achromatic petal‐shaped beams using a geometric‐phase‐based spatially multiplexed metasurface. (c) Spatially multiplexed arrangement configuration.

By designing the spatial orientation distribution of rectangular nanopillars, precise control over the wavefront of incident light can be achieved, laying the foundation for generating petal‐shaped beams with complex phase patterns. Under circularly polarized illumination, the rotationally arranged anisotropic silicon nanopillars on the metasurface provide complete 0 to 2π phase modulation based on the Pancharatnam–Berry phase mechanism. Figure 1b demonstrates how right‐handed circularly polarized (RCP) light at three wavelengths is converted into left‐handed circularly polarized (LCP) state upon transmission through the metasurface, reconstructing achromatic petal‐shaped intensity distributions in the focal plane. This geometric phase effect originates from the polarization transformation induced by the rotated nanopillars, where the phase retardation exhibits a linear relationship with twice the rotation angle *α*. Figure 1c illustrates the design methodology of the spatially multiplexed metasurface. This scheme leverages the broadband characteristics of the geometric phase to design nanopillar structures with identical periods but distinct geometrical parameters, arranged in a row‐interleaved configuration to achieve independent control at multiple wavelengths. Specifically, rows 1, 4, 7, … are assigned to the phase response at 480 nm; rows 2, 5, 8, … correspond to 530 nm; and rows 3, 6, 9, … are designated for 630 nm. Compared with conventional zone‐multiplexing methods, this approach employs a larger super‐period without degrading system performance. Instead, it enhances near‐field coupling between units operating at different wavelengths, optimizes the spatial sampling rate, and suppresses inter‐channel crosstalk by leveraging the large period and material properties. Consequently, independent wavefront control is achieved for each wavelength, enabling the generation of achromatic petal‐shaped beams with stable structural distributions. Even under triple‐wavelength multiplexing, robust performance is maintained: transmittance variation remains within 10%, and the radial distribution of the beam remains within 5%.

Figure 2a illustrates the fundamental unit cell structure of the designed metasurface, consisting of TiO_2_ rectangular nanopillars with a height of *H* = 1200 nm, periodically arranged on a silica (SiO_2_) substrate with a lattice constant of *p* = 420 nm. To achieve high‐efficiency generation of achromatic petal‐shaped beams and effectively overcome crosstalk between different structural units, a relatively large lattice constant was selected with reference to previous metasurface‐based perfect vortex beam studies. We implemented the FDTD (finite‐difference time‐domain) method to optimize the structural parameters of the nanopillars. Periodic boundary conditions (PBCs) were applied in the *x* and *y* directions to simulate a periodic array, whereas perfectly matched layers (PMLs) were applied in the propagation direction (*z*) to eliminate nonphysical reflections. The specific material and structural parameter selections are provided in the Supporting Information S1. This structure benefits from the high refractive index (*n* ≈ 2.4–2.7) and low absorption loss of titanium dioxide in the visible band, along with its excellent compatibility with manufacturing processes. Combined with the spatial multiplexing strategy and optimization of the nanopillar geometric parameters and rotation angles, it enables independent multi‐wavelength control, offering a new pathway for developing multi‐wavelength achromatic metasurfaces for petal‐shaped beams with stable geometric distributions. Here, the transmission coefficients for cross‐polarization and co‐polarization are, respectively, expressed as follows:

(5) $$\begin{array}{l} {\left|t_{\text{cross}}\right|}^{2}=\frac{1}{4}{\left|t_{x}e^{i\varphi_{x}}-t_{y}e^{i\varphi_{y}}\right|}^{2}\\ {\left|t_{\text{co}}\right|}^{2}=\frac{1}{4}{\left|t_{x}e^{i\varphi_{x}}+t_{y}e^{i\varphi_{y}}\right|}^{2} \end{array}$$

where, *t*_*x*_ and *t*_*y*_ denote the amplitude transmission coefficients for linear polarizations along the *x*‐axis and *y*‐axis, respectively, and *φ*_*x*_, *φ*_*y*_ represent the corresponding phase delays. The transmission efficiency is characterized by the ratio of the cross‐polarized transmitted energy to the total transmitted energy.

**FIGURE 2:**
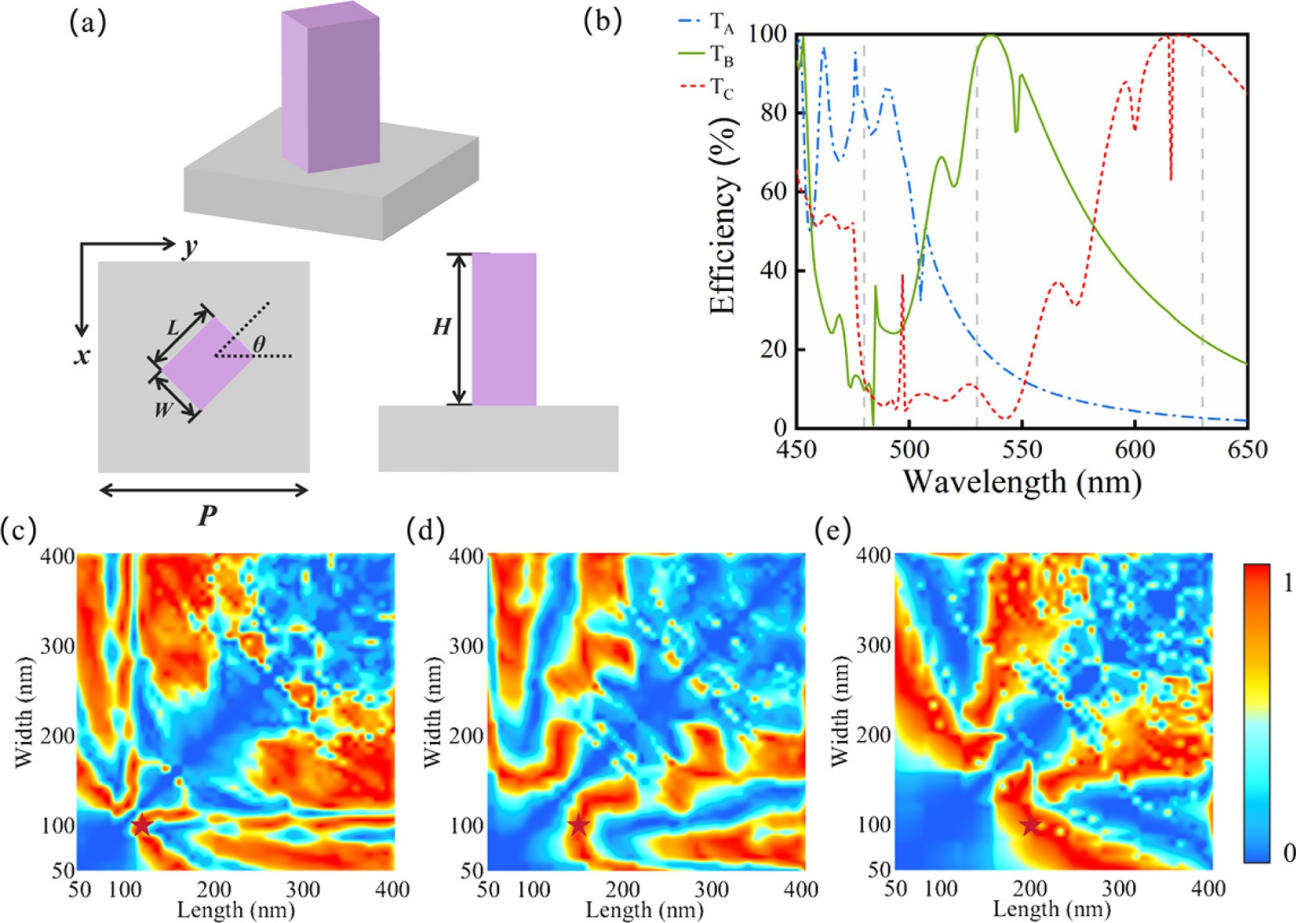
Optimization of spatially multiplexed metasurface unit cells. (a) Schematic of the TiO_2_ metasurface unit cell designed based on the Pancharatnam–Berry phase principle; (b) transmission efficiency of three unit structures with wavelength; (c–e) transmission efficiency distributions as functions of nanopillar dimensions at *λ*_A_, *λ*_B_, *λ*_C_, respectively, with star symbols marking the optimized parameter points.

As shown in Figure 2b, we performed a systematic parameter sweep to determine the optimal structural parameters of the nanopillars. For wavelengths A, B, and C, the geometric parameters of the nanopillars are *L*_A_ = 125 nm, *W*_A_ = 98 nm; *L*_B_ = 152 nm, *W*_B_ = 98 nm; and *L*_C_ = 200 nm, *W*_C_ = 98 nm, respectively. By fixing the width and varying only the length in a row‐interleaved arrangement, local high symmetry and uniform scattering cross‐section were achieved, significantly simplifying proximity effect compensation and improving critical dimension control of the nanopillar array. Additionally, this quasi‐one‐dimensional periodic arrangement provides uniform plasma distribution during etching, effectively mitigating loading effects in microfabrication. Detailed structural selection and fabrication tolerance analysis are provided in Supporting Information S1: Section S2. For these three target wavelengths, each structural unit is designed to maintain high polarization conversion efficiency (greater than 80%) only at its designated wavelength, while exhibiting low efficiency at other wavelengths. To reduce crosstalk between the different structures, the utilization efficiency for a single wavelength can reach up to 75%. Figure 2c–e present the transmission efficiency distributions as functions of nanopillar dimensions at *λ*_A_, *λ*_B_, *λ*_C_, respectively. In this work, we first selected *λ*_C_ as the central wavelength, utilizing the nearly 98% transmission efficiency of structure C at the operating wavelength to systematically investigate the generation of petal‐shaped beams with varying topological charges, sizes, and rotation angles. Subsequently, we explored dual‐wavelength (*λ*_A_ and *λ*_C_; *λ*_B_ and *λ*_C_) and triple‐wavelength (*λ*_A_, *λ*_B_, and *λ*_C_) multiplexing schemes for the generation of petal‐shaped beams based on a single all‐dielectric spatially multiplexed achromatic metasurface.

## 3 Results and Discussions

### 3.1 Generation of Petal‐Shaped Beams With Different TCs, Sizes, and Rotation Angles

Based on the preceding analysis, the generation of petal‐shaped beams is demonstrated using a 40 × 40 μm^2^ all‐dielectric metasurface with 630 nm as the central wavelength, initially through variation of TCs and sizes. The focal length was set to *f* = 80 μm, with other parameters held constant unless stated otherwise in the following. Figure 3 shows the resulting petal‐shaped beams intensity profiles for TCs of 1, 2, 3, and 4 at *NA*s of 0.15 and 0.25. In all cases, the beams display a characteristic two‐fold rotationally symmetric petal‐like pattern with uniformly distributed petals along the annular intensity ring, and the petal count is exactly twice the value of the TC. As the TC increases from 1 to 4, the annular radius remains nearly constant. For *NA* = 0.15, the measured values are 12.86 μm, 13.06 μm, 13.86 μm, and 14.06 μm, respectively; for *NA* = 0.25, these values are 22.31 μm, 22.51 μm, 22.51 μm, and 23.51 μm. These results show strong agreement with the mean radius, with all relative errors below 5%. The observed deviations are primarily attributed to discretization errors in simulation, approximations in phase generation, and numerical aperture truncation effects. Further improvements in accuracy can be achieved by refining the mesh resolution and optimizing the phase modulation model. This consistency demonstrates that the ring radius is independent of the TC of the beam, thereby confirming the geometric uniformity of the petal‐shaped beams. Meanwhile, we can see that the petal‐shaped beams radius exhibits a proportional relationship (*r* ∝ *NA*), which is consistent with theoretical predictions. It can also be found that increasing the *NA* leads to a significant expansion of the annular radius; however, the intensity distribution gradually deviates from the ideal profile. This divergence arises from two competing factors: a larger *NA* enhances the focusing capability and reduces the matching efficiency between the expanded petal‐shaped beams spot size and the finite extent of the metasurface.

**FIGURE 3:**
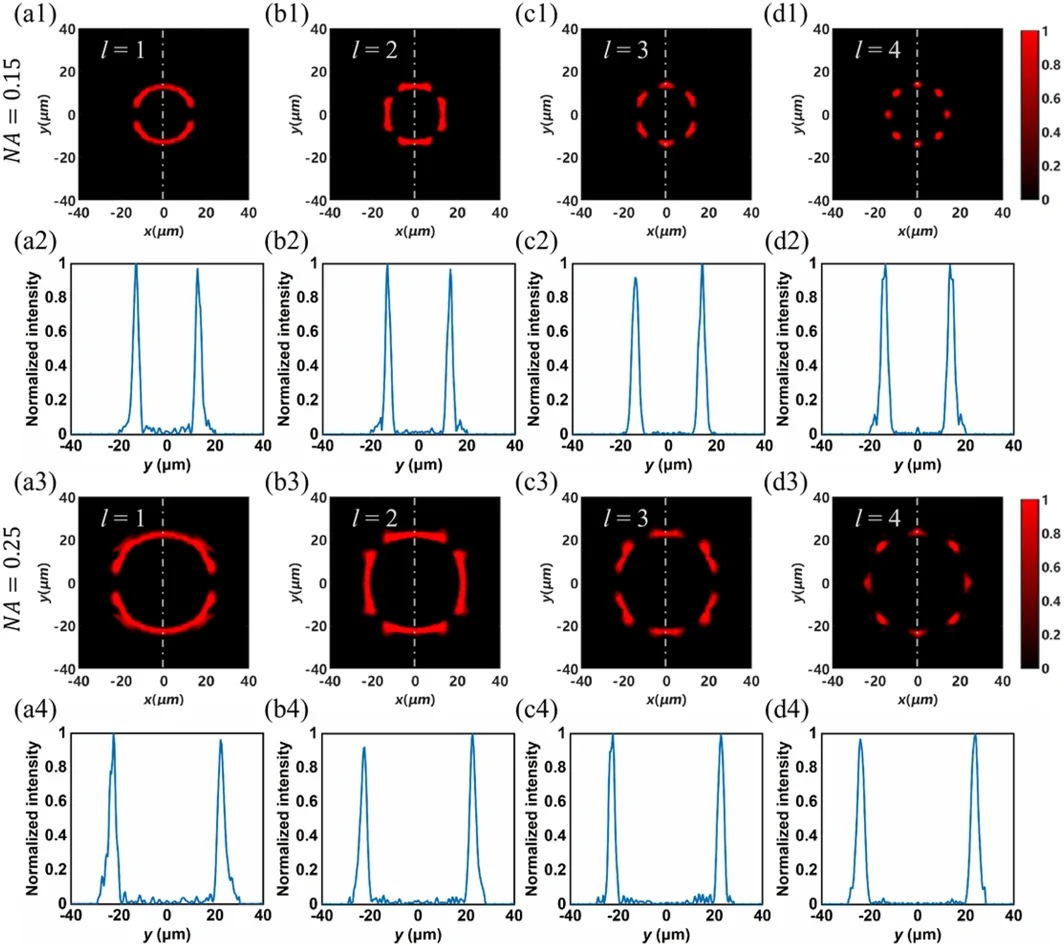
Normalized intensities in the *x–y* plane (a1–d1, a3–d3) and on vertically oriented annular plane (a2–d2, a4–d4) of petal‐shaped beams with different TCs and *NA*s based on all‐dielectric metasurface.

Based on these findings, we propose an “*NA*‐size co‐optimization” principle, with *NA* = 0.15 identified as the optimal compromise. This configuration delivers a sufficiently large ring radius (≈13 μm) for practical applications while effectively suppressing beam divergence to preserve high mode quality. Moreover, the TC can be directly and rapidly decoded from the number of petals in the intensity pattern, overcoming the limitations of conventional interferometric detection methods. By introducing the initial phase difference as an additional degree of freedom, a quantitative relationship between the phase difference and the rotational properties of the beam can be established as *α* = *δ*/(2TC), where *α* denotes the rotation angle of the beam, which is modulated by the topological charge (i.e., half the number of petals). For a fixed phase difference *δ*, a larger topological charge TC results in a smaller rotation angle *α*. Figure 4 illustrates the variation of petal‐shaped beams with respect to the rotation angle. Here, 0° is defined as the initial orientation (Figure 4a1–d1). The panels show results for different *δ* and *α*: Figure 4a2–d2 corresponds to *δ* = −2π/3, with *α* set to 60°, 30°, 20°, and 15°; Figure 4a3–d3 to *δ* = –π, with *α* = 90°, 45°, 30°, and 22.5°; and Figure 4a4–d4 correspond to *δ* = 2π/3, with *α* = −60°, −30°, −20°, and −15°. These results confirm a direct correspondence between phase difference and the rotation direction: a positive phase difference produces stable counterclockwise precession, while a negative phase difference induces controllable clockwise rotation. Based on the phase‐orientation degrees of freedom, we successfully generated petal‐shaped beams with continuously tunable rotation angles. This work establishes a foundation for designing spatially multiplexed achromatic metasurfaces, which can be applied to high‐capacity optical information transmission and coding.

**FIGURE 4:**
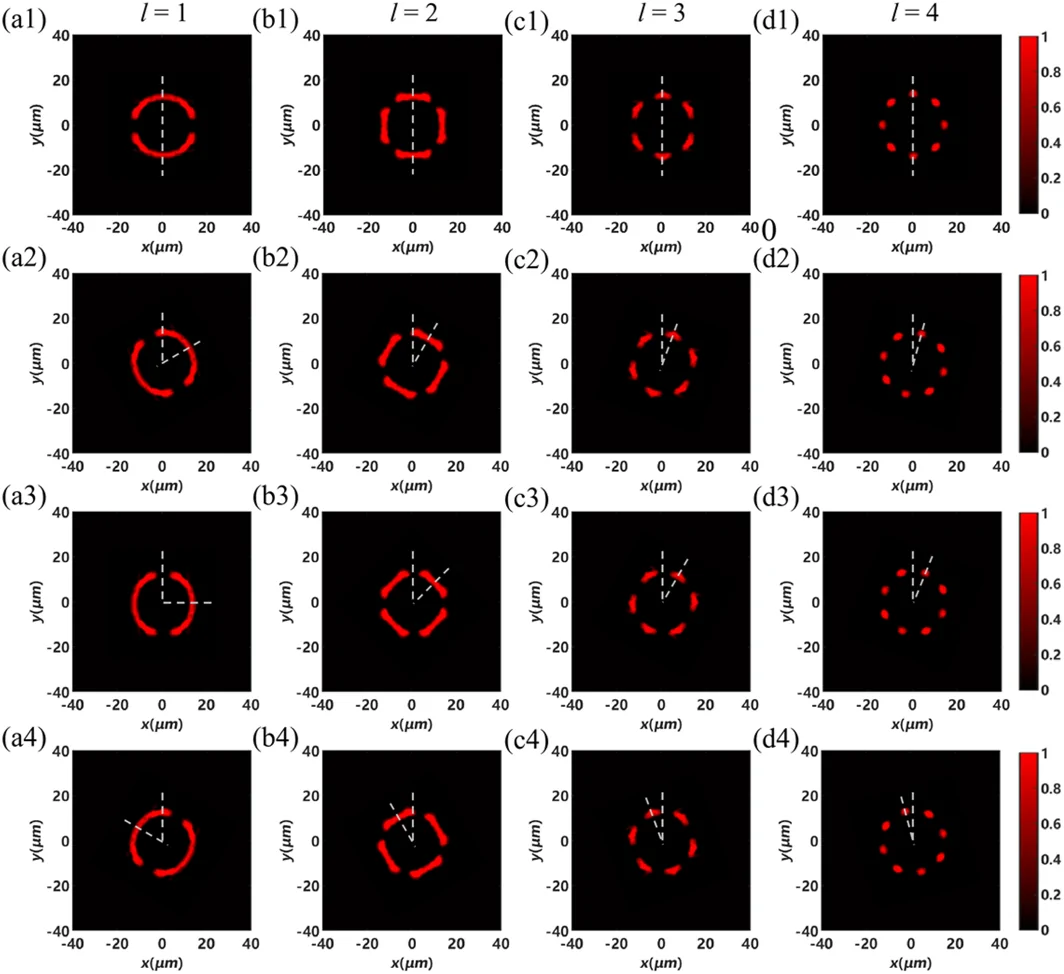
Normalized intensities distributions in the *x–y* plane of petal‐shaped beams with different rotation angles based on all‐dielectric metasurface. (a1–d1) Initial orientation; (a2–d2) *δ =* –2π/3; (a3–d3) *δ =* –π; (a4‐d4) *δ =* 2π/3.

Building on the previous work, we note that the wavelength‐dependent refractive index of the medium enables a metasurface to impart distinct phase responses to different incident wavelengths. We examined the intensity of petal‐shaped beams at operational wavelengths *λ*_A_ (Figure 5a1–c1), *λ*_B_ (Figure 5a2–c2), and *λ*_C_ (Figure 5a3–c3), finding the ring radius at design wavelength *λ*_0_ shows good agreement with theoretical predictions while significant wavelength‐dependent deviations occur at other wavelengths. Specifically, for a fixed design wavelength, any shift of the operating wavelength results in a mismatch between the actual and target ring radii of petal‐shaped beams. Additionally, the focusing behavior of petal‐shaped beams was analyzed for the case where *λ*_0_ = 480 nm. When operating at the three target wavelengths, the maximum focal position shift was observed to be approximately 12 μm (Figure 5d1–d3). This confirms that material dispersion plays a critical role in phase modulation. Consequently, compensating for the dispersion inherent in the phase response is essential to realize multi‐wavelength achromatic petal‐shaped beams. Based on a monolithic all‐dielectric spatially multiplexed metasurface, this work demonstrates achromatic petal‐shaped beam generation through dual‐wavelength and triple‐wavelength multiplexing schemes.

**FIGURE 5:**
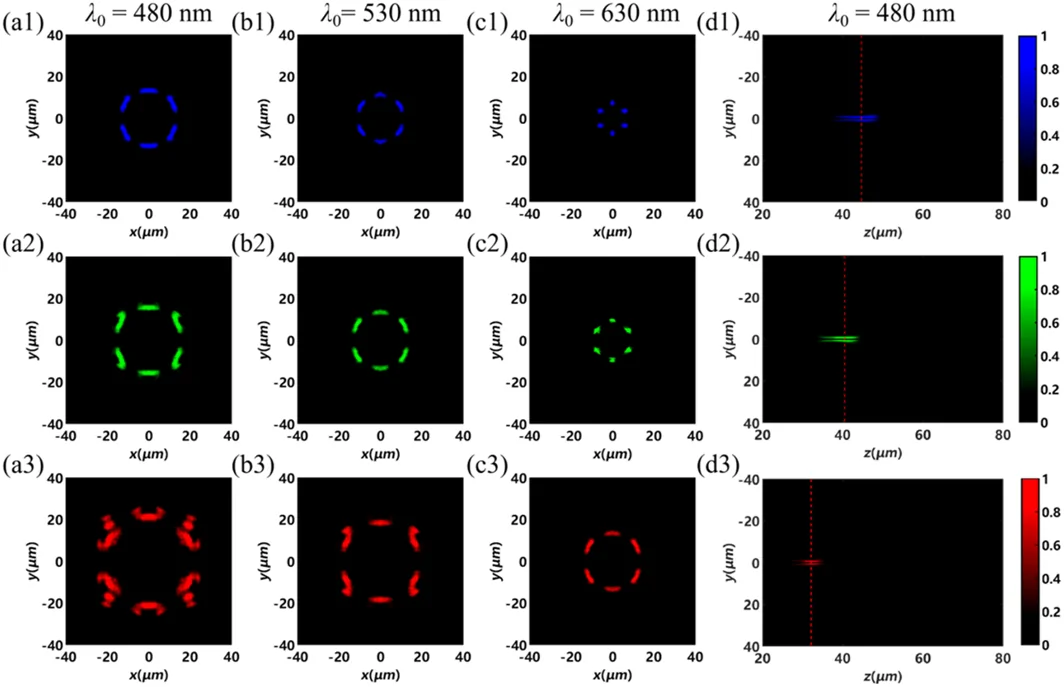
Normalized intensities distributions of petal‐shaped beams with different design wavelengths in the *x–y* plane (a1–a3) *λ*_0_ = 480 nm; (b1–b3) *λ*_0_ = 530 nm; (c1–c3) *λ*_0_ = 630 nm, along the *y–z* plane (d1–d3) *λ*_0_ = 480 nm based on all‐dielectric metasurface.

### 3.2 Design of a Multi‐Wavelength Spatially Multiplexed Achromatic Metasurface for Petal‐Shaped Beams

First, Figure 6 presents the generation of achromatic petal‐shaped beams using a dual‐wavelength spatially multiplexed metasurface at TC = 2, 4. As shown in Figure 6a1–a4, d1–d4, the beams exhibit a characteristic two‐fold rotationally symmetric petal‐like pattern with uniformly distributed petals along the annular intensity profile, where the number of petals is exactly twice the TC. The beams initially converge axially at *z* = 45 μm (Figure 6b1–b4, e1–e4) and subsequently form petal‐shaped beams with specific intensity distributions at *z* = 80 μm, with the maximum focal shifts for both metasurfaces being less than 0.6 μm. The white dashed lines mark the region of *z* = 40−50 μm, and the red dashed lines represent the focal intensity peak positions to characterize the focusing locations. For TC = 2, the measured ring radii of the petal‐shaped beams generated by the A‐C type metasurface are 12.86 and 12.86 μm, whereas those for the B‐C type are 12.86 and 12.86 μm (Figure 6c1–c4). For TC = 4, the A‐C type yields radii of 13.66 and 13.66 μm, and the B‐C type measures 13.66 and 13.66 μm (Figure 6f1–f4). The radii of petal‐shaped beams at different wavelengths remain constant, with the maximum radius variation for A‐C type and B‐C type beams under different topological charges reaching 0.8 μm. These results confirm that the ring radii of the petal‐shaped beams generated by both structures maintain high stability across different wavelengths, demonstrating very low sensitivity to wavelength variations.

**FIGURE 6:**
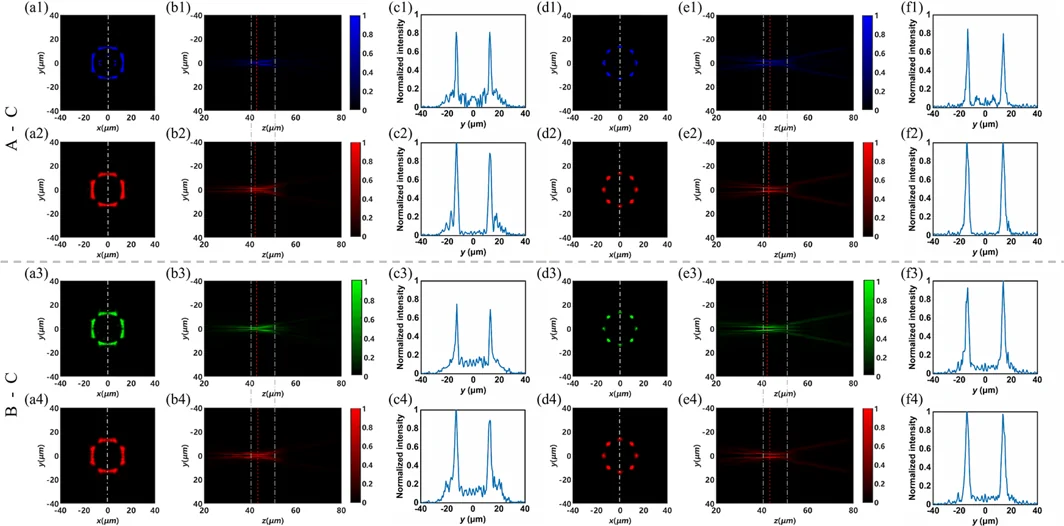
Normalized intensities in the *x–y* plane (a1–a4, d1–d4), along the *y–z* plane (b1–b4, e1–e4), and on vertically oriented annular plane (c1–c4, f1–f4) of the dual‐wavelength A–C and B–C petal‐shaped beams with TC = 2and 4 based on a single all‐dielectric metasurface.

Next, we investigate the generation of three‐wavelength achromatic petal‐shaped beams using a single spatially multiplexed metasurface for TC = 1, 2, 3, and 4 (summarized in Figure 7). At 630 nm, the measured ring radii of the petal‐shaped beams for TC = 1 to 4 are 13.46 μm, 13.86 μm, 14.06 μm, and 14.06 μm, respectively (Figure 7c3, f3, i3, l3), with a maximum variation of less than 0.6 μm, demonstrating high insensitivity to TC. For TC = 3, the ring radii at the three wavelengths are 13.46 μm, 13.86 μm, and 14.06 μm (Figure 7i1–i3), respectively, resulting in a maximum difference of 0.6 μm and a corresponding wavelength‐dependent radius coefficient of 4.35% (the wavelength dependence coefficient, defined as the ratio of the maximum radius variation to the average radius); this further confirms the remarkable wavelength independence of the structure. These results with fixed wavelength or TC collectively demonstrate that these petal‐shaped beams exhibit stable “perfect” properties. The axial intensity distributions in the *x*–*z* plane are analyzed in Figure 7h1–h3. The petal‐shaped beams first converge axially at *z =* 45 μm (with a positional deviation below 3 μm) and then form stable petal‐shaped beams at 80 μm (Figure 7g1–g3), verifying excellent achromatic performance under multi‐wavelength operation. The results demonstrate that stable petal‐shaped beam generation can be achieved while maintaining a metasurface efficiency exceeding 70%. In addition, it can be found that a higher topological charge leads to a more complex phase profile, a more uniform intensity distribution, and a darker central vortex region. For example, at TC = 4, the increased number of phase singularities enables broader spatial modulation of each vortex, contributing to a smoother phase structure and reduced local intensity nonuniformity (Figure 7j1–j3). Figure 8 summarizes the results of petal‐shaped beams generated by the multi‐wavelength achromatic metasurface, demonstrating its excellent achromatic performance and PVB‐like “perfect” characteristics through two key metrics: focal position and beam radius. Specifically, Figure 8a characterizes the focal positions (*z*) for different TCs, whereas Figure 8b presents the corresponding beam radius (*R*). Detailed results are provided in Supporting Information S1: Section S3.

**FIGURE 7:**
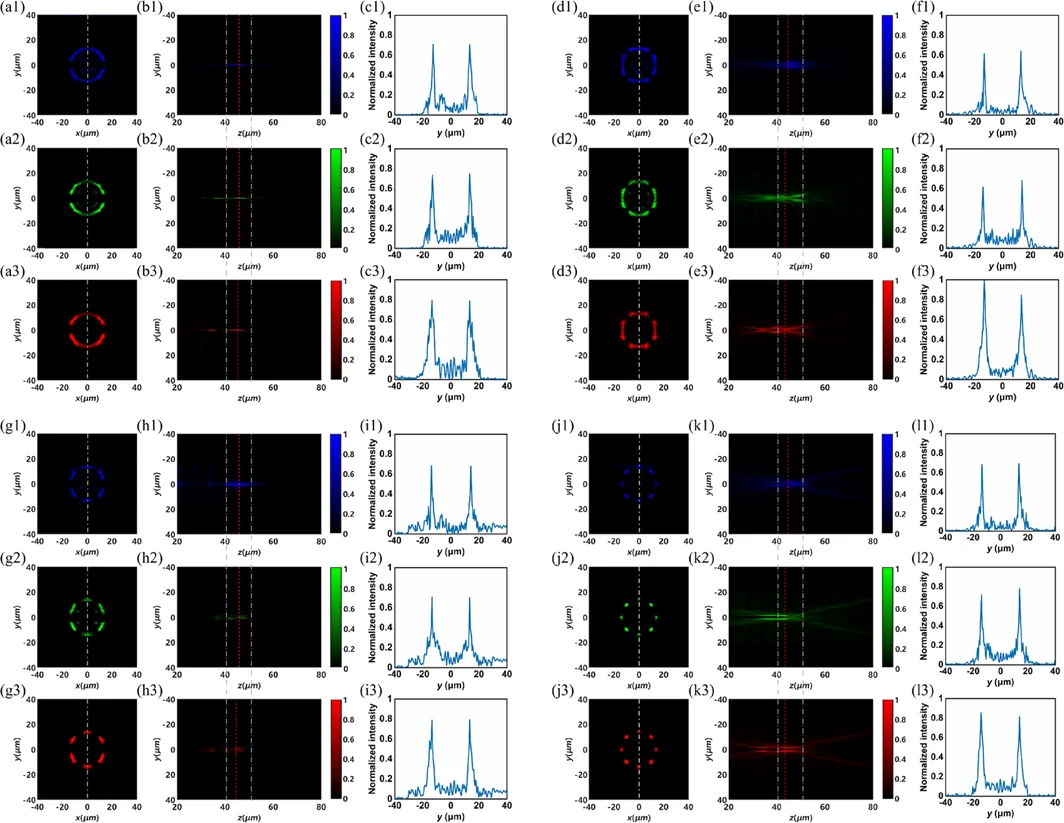
Normalized intensities in the *x–y* plane (a1–a3, d1–d3, g1–g3, and j1–j3), along the *y–z* plane (b1–b3, e1–e3, h1–h3, and k1–k3), and on vertically oriented annular plane (c1–c3, f1–f3, i1–i3, and l1–l3) of the three‐wavelength petal‐shaped beams with different TCs based on a single all‐dielectric metasurface.

**FIGURE 8:**
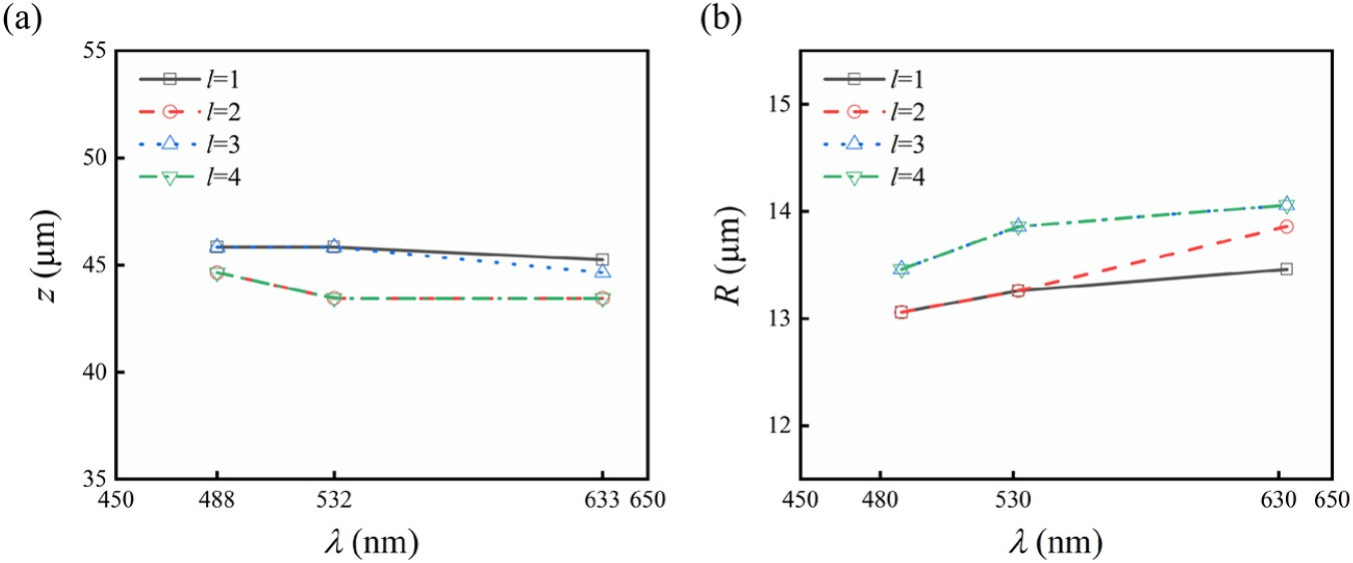
Multi‐wavelength achromatic metasurface‐generated petal‐shaped beams with different TCs. (a) Focal position. (b) Beam radius.

### 3.3 Design of a Petal‐Shaped Beam Encoding Scheme Based on Multi‐Wavelength Achromaticity

Multi‐wavelength achromatic spatially multiplexed metasurfaces enhance the stability of information transmission and further leverage the distinct rotational and intensity distribution properties of petal‐shaped beams for information encoding and encryption. We propose an encoding scheme based on the functions *N*_1_ = 36 × *M* + [4*X*+(*Y*‐1)] and *N*_2_ = 35×*M*‐[4*X*+(*Y*‐1)] for multi‐channel information encoding, where *M* takes the values 0, 1, and 1, corresponding to wavelengths *λ*_A_/*λ*_B_/*λ*_C;_ *N*_1_ and *N*_2_ represent the encoded numbers for Channel 1 and Channel 2, respectively, covering any integer from 0 to 72; *Y* denotes the TC, varying from 1 to 4 (additional degrees of freedom can be incorporated by extending the range of *Y*); and *X* corresponds to integers from 0 to 8, assigned to specific rotation angles 0°, 16°, 38°, 66°, 100°, 140°, 186°, 238°, and 296°, respectively. A partial illustration of this encoding principle is shown in Figure 9, enabling dual‐channel base‐72 and reverse base‐36 encoding on a single metasurface. Detailed encoding/decoding processes and related examples are provided in Supporting Information S1: Section S1. Compared to traditional spatial light modulators, our proposed encoding method achieves higher efficiency and integration density while fully exploiting the high‐stability characteristics of petal‐shaped beams.

**FIGURE 9:**
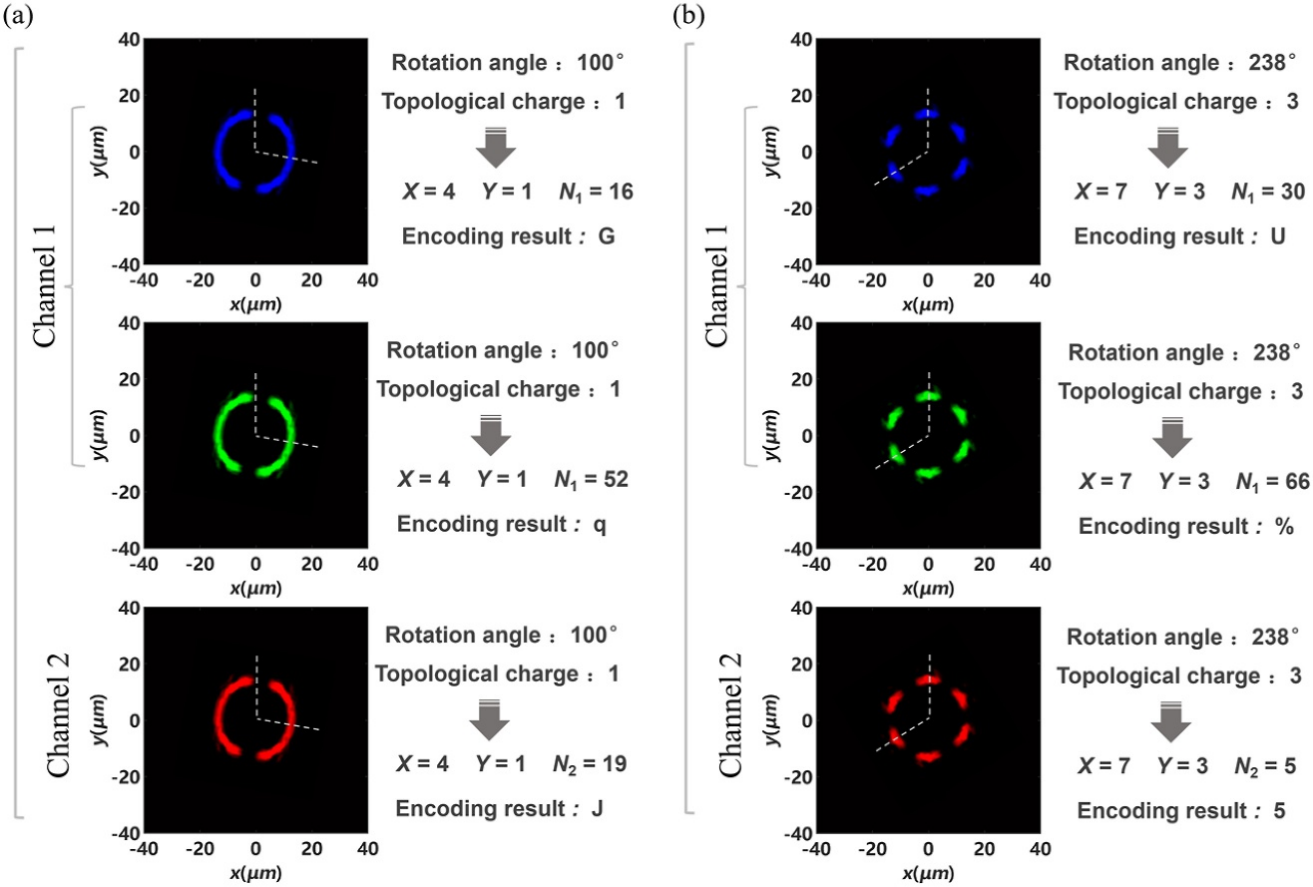
Schematic diagram of metasurface‐based multi‐wavelength petal‐shaped beam encoding. (a) TC = 1, *α* = 100°; (b) TC = 3, *α* = 238°.

## 4 Conclusion

In summary, through systematic investigation of petal‐shaped beams under various parameters, we successfully achieved the generation of petal‐shaped beams with controllable TCs, controllable radii, and tunable rotation angles. Furthermore, by leveraging a spatially multiplexed TiO_2_ metasurface design, we realized the integrated generation of achromatic petal‐shaped beams operating at multiple wavelengths. Incorporating achromatic principles, this design enables axial confocal focusing of petal‐shaped beams at distinct wavelengths (*λ* = 480 nm, 530 nm, 630 nm), effectively mitigating transmission instability induced by chromatic dispersion in conventional methods. Based on the relationships among the unique topological charge, intensity profile, and rotation angle of petal‐shaped beams, we further developed a multi‐degree‐of‐freedom optical encoding strategy. This work is anticipated to provide technical insights for next‐generation applications in multidimensional optical encryption and high‐capacity optical communications.

## Author Contributions

All authors have accepted responsibility for the entire content of this manuscript and approved its submission.

## Funding

This work was supported in part by the National Key Research and Development Program of China (2022YFA1404800), the National Natural Science Foundation of China (61975165, 62475217, 62175200); Shaanxi Provincial Natural Science Foundation (2025JC‐YBMS‐686), the Shaanxi Academy of Basic Sciences (23JSY008) and Northwestern Polytechnical University Master's Student Practice and Innovation Ability Cultivation Fund Project (PF2025077).

## Consent

Informed consent was obtained from all individuals included in this study.

## Conflicts of Interest

The authors declare no conflicts of interest.

## Supporting information


Supporting Information S1

## Data Availability

Data underlying the results presented in this paper are not publicly available at this time but may be obtained from the authors upon reasonable request.
