# Supplementary material for: Achromatic Petal‐Shaped Beam Generation and Information Encoding Based on Perfect Vortex Beam
Source: Nanophotonics. 2026 May 20;15(10):e70133. doi: 10.1002/nap2.70133 (PMC13642541; doi:10.1002/nap2.70133)
Supplement: Supplementary file 1 [file NAP2-15-e70133-s001.docx]

*Supplementary Materials for*

**Achromatic petal-shaped beam generation and information encoding based on perfect vortex beam**

*Yilong Ge, Yifeng Wei, Jinqin Cui, Ziben Liu, Yang Liu, Bingxue Zhang, Dandan Wen, Peng Li, Sheng Liu, Jianlin Zhao, and Dong Li**

Key Laboratory of Light Field Manipulation and Information Acquisition, Ministry of Industry and Information Technology, and Shaanxi Key Laboratory of Optical Information Technology, School of Physical Science and Technology, Northwestern Polytechnical University, Xi’an 710129, China,

*Corresponding author e-mail: *E-mail:* [*dongli@nwpu.edu.cn*](mailto:dongli@nwpu.edu.cn)

**1. Designing petal-shaped beams encoding scheme based on multi-wavelength achromaticity**

Multi-wavelength achromatic metasurfaces significantly enhance the stability of information transmission. The unique rotational characteristics and intensity distribution features of petal-shaped beams effectively increase the dimensionality of information, providing new possibilities for optical encoding and encryption applications.

Based on this, we propose and validate a incremental angular encoding scheme for the encoding and decoding of information in multi-wavelength, multi-channel systems.Herein, we designate wavelength *λ*_A_/*λ*_B_ as Channel 1 for base-72 information encoding, and wavelength *λ*_C_ as Channel 2 for reverse base-36 information encoding. Crucially, Channel 2 employs a reverse base-36 encoding scheme to differentiate it from Channel 1 and to serve as an effective information encryption mechanism. The multi-channel encoding function is defined as

*N*_1_=36×*M*+[4*X*+(*Y*−1)]	（S1）

*N*_2_=35×*M*-[4*X*+(*Y*−1)]	（S2）

Where, *N*_1_ and *N*_2_ represent the dual-channel encoded numbers for base-72 and base-36 encoding, respectively; *Y* denotes the topological charge value, ranging from 1 to 4 (the degree of freedom can be further increased by raising the TC value); *X* corresponds to the rotation angle index, ranging from 0 to 8. *M* corresponds to the multi-wavelength configuration: it takes a value of 0 for *λ*_A_, and a value of 1 for *λ*_B_/*λ*_C_. The initial value of *α* is 0°, and it increments with a starting step of 16° according to the following rule

*α_X_*=0°, 16°, 38°, 66°, 100°, 140°, 186°, 238°, 296°；

Its recurrence formula is

*α_X_*=*α_X_*_−1_+16+6×(*X*−1)	（*X*≥1）	（S3）

The purpose of selecting an increasing angle is to eliminate the influence of symmetric intensity distribution on petal-shaped beams angle recognition. The angle mapping relation is shown in Figure S1.

**Figure S1:** Encoded angle mapping. Digits 0-8 correspond to the following angles, respectively: 0°, 16°, 38°, 66°, 100°, 140°, 186°, 238°, 296°.

Here, we present the specific encoding and decoding procedure as follows

For *λ*_A_ , when *M*=0, *X* = 0, *Y* = 1, *N*_1_ = 0; *Y* = 2, *N*_1_ = 1; *Y* = 3, *N*_1_ = 2; *Y* = 4, *N*_1_ = 3.

When *M*=0, *X* = 1, *Y* = 1, *N*_1_ = 4; *Y* = 2, *N*_1_ = 5; *Y* = 3, *N*_1_ = 6; *Y* = 4, *N*_1_ = 7.

For *λ*_B_ , when *M*=1, *X* = 8, *Y* = 1, *N*_1_ = 68; *Y* = 2, *N*_1_ = 69; *Y* = 3, *N*_1_ = 70; *Y* = 4, *N*_1_ = 71.

For *λ*_C_ , when *M*=1, *X* = 8, *Y* = 1, *N*_1_ = 3; *Y* = 2, *N*_2_ = 2; *Y* = 3, *N*_2_ = 1; *Y* = 4, *N*_2_ = 0.

Decoding method is defined as

*X*=[(*N*_1_*−*36×*M*)/4]; *Y*=[(*N*_1_*−*36×*M*)mod4]+1	（S4）

*X*=[(35×*M−N*_2_)/4]; *Y*=[(35×*M−N*_2_)mod4]+1	（S5）

For *λ*_A_ , *N*_1_=33; *M*=0;

*X*=[(*N*_1_−0)/4]=8;

*Y*=[(*N*_1_−0)mod4]+1=1+1=2;

For *λ*_B_ , *N*_1_=55; *M*=1;

*X*=[(*N*_1_−36)/4]=4;

*Y*=[(*N*_1_−36)mod4]+1=3+1=4;

For *λ*_C_ , *N*_2_=28; *M*=1;

*X*=[(35−*N*_2_)/4]=1;

*Y*=[(35−*N*_2_)mod4]+1=4;

*N*_2_=0; *M*=1;

*X*=[(35−*N*_2_)/4]=8;

*Y*=[(35−*N*_2_)mod4]+1=4;

Using the encoding method described above, the dual-channel base-72 plus base-36 scheme enables the encryption and transmission representation of information. Table S1 displays the correspondence of encoded numbers, and Figure S2 provides a schematic diagram of the corresponding petal-shaped beam encoding. It is worth noting that for the final ten encoded characters of the base-72 scheme, they strictly follow the order of the keyboard symbol section. Table S2 illustrates the specific encoding representation, presenting the mapping of encoded numbers for the dual-channel base-72 plus base-36 scheme.

Table S1: Encoding scheme diagram

| Digital | Represents content | Meaning |
| --- | --- | --- |
| 0-9 | Digits 0-9 | Direct mapping of digits |
| 10-35 | Letters A-Z | 10=A 11=B...34=Y 35=Z |
| 36-61 | Letters a-z | 36=a 37=b...60=y 61=z |
| 62-71 | ! @ # $ % ^ & * ( ) | Specific characters |

**Figure S2:** Schematic diagram of petal-shaped beam encoding.

Table S2: Encoding map

| *X* | *Y* | *Y*-1 | *N*_1_ (*λ*_A_/*λ*_B_) | Channel 1 | *N*_2_ (*λ*_C_) | Channel 2 |
| --- | --- | --- | --- | --- | --- | --- |
| 0 | 1 | 0 | 0 /36 | 0 /a | 35 | Z |
| 0 | 2 | 1 | 1 /37 | 1 /b | 34 | Y |
| 0 | 3 | 2 | 2 /38 | 2 /c | 33 | X |
| 0 | 4 | 3 | 3 /39 | 3 /d | 32 | W |
| 1 | 1 | 0 | 4 /40 | 4 /e | 31 | V |
| 1 | 2 | 1 | 5 /41 | 5 /f | 30 | U |
| 1 | 3 | 2 | 6 /42 | 6 /g | 29 | T |
| 1 | 4 | 3 | 7 /43 | 7 /h | 28 | S |
| 2 | 1 | 0 | 8 /44 | 8 /i | 27 | R |
| 2 | 2 | 1 | 9 /45 | 9 /j | 26 | Q |
| 2 | 3 | 2 | 10 /46 | A /k | 25 | P |
| 2 | 4 | 3 | 11 /47 | B /l | 24 | O |
| 3 | 1 | 0 | 12 /48 | C /m | 23 | N |
| 3 | 2 | 1 | 13 /49 | D /n | 22 | M |
| 3 | 3 | 2 | 14 /50 | E /o | 21 | L |
| 3 | 4 | 3 | 15 /51 | F /p | 20 | K |
| 4 | 1 | 0 | 16 /52 | G /q | 19 | J |
| 4 | 2 | 1 | 17 /53 | H /r | 18 | I |
| 4 | 3 | 2 | 18 /54 | I /s | 17 | H |
| 4 | 4 | 3 | 19 /55 | J /t | 16 | G |
| 5 | 1 | 0 | 20 /56 | K /u | 15 | F |
| 5 | 2 | 1 | 21 /57 | L /v | 14 | E |
| 5 | 3 | 2 | 22 /58 | M /w | 13 | D |
| 5 | 4 | 3 | 23 /59 | N /x | 12 | C |
| 6 | 1 | 0 | 24 /60 | O /y | 11 | B |
| 6 | 2 | 1 | 25 /61 | P /z | 10 | A |
| 6 | 3 | 2 | 26 /62 | Q /! | 9 | 9 |
| 6 | 4 | 3 | 27 /63 | R /@ | 8 | 8 |
| 7 | 1 | 0 | 28 /64 | S /# | 7 | 7 |
| 7 | 2 | 1 | 29 /65 | T /$ | 6 | 6 |
| 7 | 3 | 2 | 30 /66 | U /% | 5 | 5 |
| 7 | 4 | 3 | 31 /67 | V /^ | 4 | 4 |
| 8 | 1 | 0 | 32 /68 | W /& | 3 | 3 |
| 8 | 2 | 1 | 33 /69 | X /* | 2 | 2 |
| 8 | 3 | 2 | 34 /70 | Y /( | 1 | 1 |
| 8 | 4 | 3 | 35/71 | Z/) | 0 | 0 |

**2. Based on the material selection for achromatic metasurfaces in this study, we considered two commonly used materials**

(1) Silicon (Si), which has a high refractive index (*n*≈4.3). For the spatial multiplexing achromatic principle being validated, the design requires nanofins with different optical responses to be closely interleaved at subwavelength scales. According to Mie resonance theory, the effective mode volume of the resonant field is inversely proportional to the refractive index. The high refractive index of Si tightly confines the optical field within each nanofin, effectively isolating crosstalk between adjacent structures. However, Si exhibits significant absorption loss in the visible band, particularly at the 480 nm wavelength.

Consequently, the overall transmission efficiency distribution as a function of nanopillar dimensions for wavelengths *λ*_A_, *λ*_B_, and *λ*_C_ is relatively broad and dispersed. This provides some flexibility in selecting individual unit cell responses but also results in a generally lower overall metasurface generation efficiency.

(2) Titanium Dioxide (TiO_2_) possesses a relatively lower refractive index (*n*≈2.4). To achieve a full 0–2π phase coverage, unit structures with high aspect ratios are typically required. However, TiO_2_ exhibits low absorption loss in the visible band and provides higher transmission. As a result, for TiO_2_ metasurfaces, the overall transmission efficiency distribution as a function of nanopillar dimensions for wavelengths *λ*_A_, *λ*_B_, and *λ*_C_ tends to be more concentrated and uniform during parameter sweeps, which introduces some difficulty in selecting a single, optimal unit structure.

Figure S3 presents the polarization conversion efficiency distributions for wavelengths *λ*_A_, *λ*_B_, and *λ*_C_ under the same structural conditions (*P*=400 nm, *H*=800 nm) for both materials: Figure S3(a1–c1) corresponds to Silicon (Si), and Figure S3(a2–c2) corresponds to Titanium Dioxide (TiO_2_). Comparing these two materials, and considering the impact of metasurface generation efficiency on petal-shaped beams, where the average efficiency for Si is approximately 30%, while for TiO_2_ it can reach approximately 70%—along with an improvement in beam stability, this study prioritizes TiO_2_ as the primary material for the metasurface unit cells.

**Figure S3:** Transmission efficiency distributions as functions of nanopillar dimensions at *λ*_A_, *λ*_B_, *λ*_C_ of different materials_。_(a1-c1) Si; (a2-c2) TiO_2_.

We also considered the influence of unit cell height on metasurface generation efficiency. Figure S4 presents the polarization conversion efficiency distributions for a TiO_2_ metasurface at wavelengths *λ*_A_, *λ*_B_, and *λ*_C_ with a period *P*=420 nm, for unit cell heights of 1000 nm and 1200 nm. Specifically, Figure S4(a1–c1) corresponds to *H*=1000 nm, and Figure S4(a2–c2) corresponds to *H*=1200 nm. A clear observation is that increasing the unit cell height has a positive impact on polarization conversion efficiency. It is particularly noteworthy that the 420 nm period design effectively increases the spacing between nanopillars of different structures. This enlargement helps reduce crosstalk among multi-wavelength optical signals, thereby optimizing device performance.

**Figure S4:** Transmission efficiency distributions as functions of nanopillar dimensions at *λ*_A_, *λ*_B_, *λ*_C_ for unit cells with different heights._。_(a1-c1) *H*=1000; (a2-c2) *H*=1200.

In addition, considering the impact of fabrication tolerances on practical applications, this work selected the following unit cell parameters for the three wavelengths *λ*_A_, *λ*_B_, and *λ*_C_: *L*_A_=125 nm, *W*_A_=98 nm; *L*_B_=152 nm, *W*_B_=98 nm; and *L*_C_=200 nm, *W*_C_=98 nm; *P*=420nm; *H*=1200nm. Notably, we intentionally kept the width constant at 98 nm. This ensures optimal uniformity in the sidewall angle, etch depth, and width control across all nanopillars on the actual metasurface during fabrication. If different lengths and widths were used, structures that are wider or narrower would respond differently to the etching conditions, potentially causing certain dimensions to deviate from the design values and thereby compromising the accuracy of the phase response. Fixing the width fundamentally eliminates this variable.

To make the validation framework more rigorous and comprehensive, we performed a full tolerance sensitivity analysis of the metasurface with respect to key geometric parameters—length, width, and height—using transmission efficiency (defined as the ratio of cross-polarized transmitted energy to total transmitted energy) as the core metric:

(1) When the lateral dimensions (*L*, *W*) fluctuate within a range of ±5 nm, the phase response transmission efficiency for each individual wavelength (480 nm, 530 nm, and 630 nm) remains above 85%, while the transmission efficiency at the other wavelengths remains low. This satisfies the design requirements for unit cells in spatially multiplexed metasurfaces. It is verified that the radial distribution, longitudinal focusing, and geometric fidelity of the generated petal-shaped beams remain stable, with errors kept within 5%.
(2) When the vertical dimension (*H*) fluctuates within a range of 20 nm, the phase response transmission efficiency for each individual wavelength (480 nm, 530 nm, and 630 nm) remains above 95%, while the transmission efficiency at the other wavelengths remains low. This again meets the design requirements for unit cells in spatially multiplexed metasurfaces. Notably, the effect of height variation on generation efficiency is relatively small, which is primarily attributed to the fact that geometric-phase metasurfaces are mainly influenced by the lateral geometric dimensions of the unit structures. It is verified that the radial distribution, longitudinal focusing, and geometric fidelity of the generated petal-shaped beams remain stable, with errors kept within 2%.

Table S3: Fabrication Tolerance Analysis (Transmission Efficiency as a Function of *L*, *W*, *H*)

| *λ* | 480 nm | 530 nm | 630 nm |
| --- | --- | --- | --- |
| *L W H* | 125 nm 98 nm 1200 nm | 152 nm 98 nm 1200 nm | 200 nm 98 nm 1200 nm |
| *L* -5 nm  +5 nm | 94%  85% | 95%  93% | 94%  99% |
| *W* -5 nm  +5 nm | 96%  98% | 99%  99% | 97%  99% |
| *H* -5 nm  +5 nm | 97%  99% | 98%  99% | 97%  99% |

These results demonstrate that the designed unit cell structures exhibit strong robustness against non-ideal fabrication morphologies. The quantification of the impact of manufacturing errors on device performance provides a solid guarantee for the engineering feasibility and optical performance consistency of the proposed approach within current micro-/nano-fabrication precision limits.

Meanwhile, although the larger structural height improves the overall polarization conversion efficiency of the metasurface, we also considered the challenges that greater height poses for the manufacturing process. Therefore, for the wavelengths 488 nm, 532 nm, and 633 nm, we specifically determined the unit cell parameters under the condition of height *H*=800 nm to facilitate subsequent experimental research. These parameters are: *L*_A_=136 nm, *W*_A_=95 nm; *L*_B_=220 nm, *W*_B_=160 nm; and *L*_C_=340 nm, *W*_C_=110 nm; *P*=400nm; *H*=800nm. Figure S5 presents the corresponding transmission efficiency of the three unit structures as a function of wavelength. This configuration ensures that all polarization conversion efficiencies exceed 80%.

**Figure S5:** Transmission efficiency of the three unit structures as a function of wavelengths 488 nm, 532 nm, and 633 nm

**3.** **Geometric and focusing distribution characteristics of petal-shaped beams under different wavelengths and topological charges.**

Benefiting from the low absorption loss of TiO_2_ in the visible band and the high transmission efficiency enabled by its high-aspect-ratio structure, the designed metasurface exhibits excellent light-field manipulation capabilities. Table S4 and Table S5 present detailed results of the geometric and focusing distributions of petal-shaped beams under different wavelengths and topological charges, respectively.

Table S4: Focal positions of petal-shaped beams (TiO_2_) with different TCs (unit: μm)

|  | TC=1 | TC=2 | TC=3 | TC=4 |
| --- | --- | --- | --- | --- |
| *λ*_A_ | 45.84 | 44.65 | 45.84 | 44.65 |
| *λ*_B_ | 45.84 | 43.45 | 45.84 | 43.45 |
| *λ*_C_ | 45.25 | 43.45 | 44.65 | 43.45 |

Table S5: Beam radius of petal-shaped beams (TiO_2_) with different TCs (unit: μm)

|  | TC=1 | TC=2 | TC=3 | TC=4 |
| --- | --- | --- | --- | --- |
| *λ*_A_ | 13.06 | 13.06 | 13.46 | 13.46 |
| *λ*_B_ | 13.26 | 13.26 | 13.86 | 13.86 |
| *λ*_C_ | 13.46 | 13.86 | 14.06 | 14.06 |
